# Oxidation of Methionine-35 in Alzheimer’s amyloid-beta peptide and the aggregation of the oxidized peptide

**DOI:** 10.1186/2193-1801-4-S1-P13

**Published:** 2015-06-12

**Authors:** Merlin Friedemann, Eneken Helk, Ann Tiiman, Kairit Zovo, Peep Palumaa, Vello Tõugu

**Affiliations:** Tallinn University of Technology, Estonia

**Keywords:** β-amyloid, copper(II)ion, methionine oxidation

Alzheimer’s disease (AD) is a neurodegenerative disease characterized by progressive loss of brain tissue and accumulation of amyloid-β(Aβ) and tau. Aggregation of Aβ peptides into amyloid plaques is considered to be a causative factor in AD, however the precise mechanism behind the AD onset has remained elusive. Oxidative stress (OS) is also characteristic to AD, but it is not known whether OS is a risk factor or a consequence of AD. It is assumed that Aβ aggregates generate free radicals in the presence of copper ions by participation of the Met35 residue, which can increase the OS levels. The aim of our study was to establish the role of Met35 residue in the oxidation of Aβ and peptide aggregation processes. Oxidation of Aβ was studied in the presence of two redox-active compounds: H2O2 and copper ions. In the absence of copper ions the Met35 residue was readily oxidized by H2O2 in a two electron process. The fibrillization of Aβ with Met35 oxidized to sulfoxide was threefold slower compared to that of the native peptide. TEM analysis showed that the fibrils of native and oxidized peptides are similar. The relatively small inhibitory effect of Met35 oxidation on the fibrillization suggests that the possible variation in the Met oxidation state should not affect the in vivo plaque formation. In the presence of copper ions (one-electron process) the oxidation was more complex: addition of the first oxygen was still the fastest process, however, it was accompanied by multiple unspecific modifications of several amino acid residues. Addition of copper ions to the already oxidized Aβ Met35 by H2O2, resulted in a similar pattern of nonspecific modifications, suggesting that the one-electron oxidation processes in Aβ do not depend on the oxidation state of Met35. Thus, it can be concluded that Met35 residue is not a part of the radical generating mechanism of Aβ-Cu(II) complex.

